# Editorial: Artificial intelligence and robotic applications for smart monitoring and assistance in healthcare services

**DOI:** 10.3389/frobt.2024.1527773

**Published:** 2024-12-02

**Authors:** Ciro Mennella, Umberto Maniscalco, Giovanni Luca Masala, Massimo Esposito

**Affiliations:** ^1^ Institute for High-Performance Computing and Networking (ICAR) Research National Council of Italy (CNR), Naples, Italy; ^2^ School of Computing, University of Kent, Canterbury, United Kingdom

**Keywords:** deep learning, machine learning, assistive systems, health monitoring, AI-powered decision making, digital therapeutics

Emerging technological advances have attracted considerable interest within the healthcare research community. In the literature, a wealth of studies continues to emerge, emphasizing innovative strategies and technologies designed for healthcare applications. Recent research efforts are increasingly investigating methodologies and techniques that utilize data-driven and personalized approaches to improve diagnosis, treatment, and therapy. In particular, artificial intelligence (AI) and robotics systems are considered to potentially revolutionize healthcare by offering new solutions to enhance decision-making and empower clinical assistance.

Considering this rapidly evolving research on AI and robotic systems, this Research Topic was proposed to collect the ongoing efforts and insights by the research community in the healthcare field. Particularly, the Research Topic aims to collect innovative contributions in advancing monitoring and assistance systems for clinical applications.

A total of 5 contributions were selected for publication within this Research Topic. Each article showcased innovative solutions that can support clinical assistance, improve decision-making, and ultimately lead to better patient outcomes. Together, these studies reveal diverse applications that target specific healthcare challenges, from improving developmental monitoring in infants and enhancing mobility for adolescents with neurological impairments to optimizing diabetic retinopathy, advancing brain-computer interfaces, and refining patient-robotic interaction simulation data.

Below is presented a summary of the contributions included in this Research Topic.

Udayagiri et al. presented *“Towards an AI-driven soft toy for automatically detecting and classifying infant-toy interactions using optical force sensors.”* This study introduces an innovative soft toy equipped with optical force sensors designed to automatically detect and classify various infant-toy interactions. By analyzing interaction patterns, the toy can potentially identify infants at risk of neurodevelopmental delays, enabling early intervention. The machine learning model developed for this instrumented toy demonstrates promising accuracy in recognizing distinct types of interactions, paving the way for broader applications in developmental monitoring and early diagnosis.

Basla et al. contributed *“Enhancing walking efficiency of adolescents with neurological impairments using an exosuit for ambulatory activities of daily living.”* This research explores the use of a robotic suit, to improve walking efficiency in adolescents with neurological impairments. Although the results were not statistically significant, participants showed reduced time and step counts with the exoskeleton assistance, suggesting that such wearable technology could enhance functional independence and participation in daily activities. However, the authors note that further design modifications are necessary to improve practicality and ease of use.

Polyakov et al. presented *“Recruiting neural field theory for data augmentation in a motor imagery brain-computer interface.”* This article proposes a unique approach using neural field theory (NFT) to augment training data by generating artificial electroencephalogram (EEG) signals, enhancing classification accuracy for motor imagery tasks in brain-computer interfaces (BCIs). By addressing the limitations of limited training datasets, this method leverages biophysically accurate artificial data to improve BCI performance, marking a significant step forward in the future development of adaptive healthcare interfaces.

Röhl et al. contributed *“Effect of simulated hearing loss on automatic speech recognition for an android robot-patient.”* This study addresses the need to improve robot-patient interaction in medical training assessment applications by accurately simulating patient behaviors under hearing loss conditions. By evaluating automatic speech recognition systems under these simulated conditions, the study provides insights into how hearing impairments affect speech recognition performance in robotic systems. These findings highlight the importance of incorporating realistic patient behavior data into robotic simulation data, which can improve the effectiveness of the system in real-world challenges.

Alam et al. presented *“SwAV-driven diagnostics: new perspectives on grading diabetic retinopathy from retinal photography.”* This study focuses on diabetic retinopathy and introduces a novel algorithm, Swapping Assignments between multiple Views (SwAV), to improve grading accuracy from retinal images. The developed approach outperforms state-of-the-art Convolutional Neural Network (CNN) and Transformer-based models in terms of accuracy, efficiency, and computational cost, making it a promising tool for early detection and intervention in diabetic retinopathy with potential clinical application.

The Research Topic of research presented here underscores the impact that advanced technologies, especially AI and robotic applications, can have in revolutionizing healthcare systems. Advanced technologies, for instance, will not only contribute to enhancing clinical decision-making but also contribute to offering a more patient-centered and preventive approach to care. As these tools continue to evolve, they promise a more efficient, responsive, and data-driven healthcare ecosystem. However, to maximize their impact and mitigate potential risks, all stakeholders must address Research Topic related to data privacy, ethical AI applications, and equitable access to ensure the responsible and effective implementation of these technologies.

The works presented in this editorial represent early steps toward a future where these technologies are fully integrated into routine healthcare practice. However, realizing this future will require continued research, cross-disciplinary collaboration, and thoughtful integration of new tools into clinical workflows. Future efforts are needed from researchers across clinical and engineering fields to improve the integration of technological advancements and facilitate the transfer of these innovations into clinical practice. Cross-disciplinary collaboration will be essential for refining proof-of-concept tools and ensuring their practical application in healthcare.

